# Large neighborhood search and hyper-heuristics for the capacitated p-median problem

**DOI:** 10.1007/s10732-025-09580-3

**Published:** 2026-01-26

**Authors:** Ida Gjergji, Lucas Kletzander, Nysret Musliu

**Affiliations:** 1 DBAI, TU Wien, Favoritenstrasse 11, Vienna, 1040 Austria; 2 Christian Doppler Laboratory for AI and Optimization for Planning and Scheduling, DBAI, TU Wien, Favoritenstrasse 11, Vienna, 1040 Austria

**Keywords:** Meta-heuristics, Hyper-heuristics, p-median problem, Discrete optimization, Low-level heuristics

## Abstract

The p-median problem has a central importance in the context of location planning problems. An extended version of this problem is the capacitated p-median problem (CPMP) which is used for diverse applications in urban planning and medical care units location. Given its relevance and its practicality, in this paper we present a large neighborhood search (LNS) and a study of hyper-heuristics for the CPMP. We propose and analyze various destruction operators within the framework of LNS to efficiently explore diverse neighborhoods. An exact solver is used in the repair phase. Additionally, these operators are also used for the hyper-heuristics, which are high-level problem-independent solution approaches, to propose new low-level heuristics. We provide a comparison of the LNS and of the best performing hyper-heuristics with state-of-art approaches for this problem. The proposed solution methods provide a lower average GAP value compared to the state of the art and find better solutions for several instances. We also give a detailed analysis of the performance of the low-level heuristics and their impact on the instances with different structure. These results confirm the robust performance of hyper-heuristics for the CPMP, which have the advantage to allow the employment on similar scenarios with minor adjustments.

## Introduction

The p-median problem (PMP) is a location-allocation problem that involves a set of demand points and a set of candidate locations that can offer service to these demand points. The objective is to come up with a solution that gives the minimum total distance among the *p* selected locations as service deliverers and their assigned demand points. The PMP has been extensively used in various application scenarios. An example would be in urban and regional planning where Mu and Tong (2018) present an algorithm for the PMP as a possibility to be included in Geographic Information System (GIS) packages for distinct location-allocation problems. Another application of PMP is its use for the location of ambulances in Perth city, Australia by Dzator and Dzator (2013). Other use cases are reported in supply chain design (Daskin et al. 2005), in mobile communication networks (Salcedo-Sanz et al. 2008), and in decision-making (Tajbakhsh and Shamsi 2019). Typically the candidate locations have some limitations in terms of the service they can offer. Therefore, we focus our attention on the capacitated version of the p-median problem (CPMP), where candidate medians have determined capacities.

This problem is an NP-hard problem (Garey and Johnson 1979), and various methods have been proposed in the literature. Starting with exact methods, a set partitioning formulation of the CPMP was studied by Baldacci et al. (2002). A column generation method for the CPMP has been introduced by Lorena and Senne (2004). CPMP has also been tackled with a branch-and-price algorithm by Ceselli and Righini (2005). Another exact method is the cutting plane algorithm constructed upon Fenchel cuts by Boccia et al. (2008).

Continuing with heuristic approaches, Scheuerer and Wendolsky (2006) presented a scatter search technique for the capacitated clustering problem. Fleszar and Hindi (2008) introduced a variable neighborhood search for the CPMP. A genetic algorithm was proposed by Oksuz et al. (2022).

Lastly, several hybrid techniques have been presented for the CPMP. Osman and Christofides (1994) proposed a hybrid technique composed of the acceptance criterion of simulated annealing and tabu search. A greedy random adaptive memory search has been introduced by Ahmadi and Osman (2005) for the CPMP. Díaz and Fernandez (2006) developed a hybrid approach based on scatter search and path relinking. Additionally, Chaves et al. (2007) proposed a heuristic based on cluster search. Yaghini et al. (2013) presented a hybrid metaheuristic that integrates tabu search with cutting-plane neighborhood. A matheuristic was proposed by Stefanello et al. (2015), followed by another matheuristic by Gnägi and Baumann (2021).

Although state-of-the-art methods usually provide good solutions for the available instances in the literature, the optimal solutions for many instances are not known. Therefore, the study of new methods in this domain remains crucial. Moreover, the investigation and evaluation of problem-independent solving techniques for this problem is of high interest, as such methods can be used for related problems with less effort, by just providing basic solution components. In particular, in this paper we focus on large neighborhood search and hyper-heuristics approaches.

Large neighborhood search (LNS) (Shaw 1998) is a robust local search approach that represents state of the art in several optimization problems. By means of destroy and repair operators, the LNS procedure can help to find better solutions in every step of the algorithm. The design of the composing operators depends on the problem domain for which the implementation is targeted.

Hyper-heuristic techniques are an example of high-level, problem-independent approaches. The aim of hyper-heuristics is to automate the design of heuristic methods based on a combination of low-level heuristics. Hyper-heuristics can be thought of as ’heuristics to choose heuristics’ (Burke et al. 2013). The main idea here is that, with a set of low-level heuristics (LLH), the hyper-heuristics guide the combination of LLHs to provide good solutions for diverse problem domains (Burke et al. 2013, 2019).

One of the differences between LNS and hyper-heuristics is that the LNS is structured based on the characteristics of the problem under investigation unlike hyper-heuristics. Hyper-heuristics operate on the ’heuristics’ space whilst LNS works on the solution space.

This paper is a new contribution based on the conference paper presented in MIC24 (Gjergji and Musliu 2024) where a large neighborhood search was proposed. The extension includes a deep investigation of hyper-heuristics for the CPMP, which to the best of our knowledge have not been considered earlier for this problem. The main contributions of this paper are:

- We investigate a LNS method with different destroy operators and use a MIP solver in the repair stage.
- We study the impact of different operators in the performance of LNS.
- We propose several novel low-level heuristics and an initial solution method for the CPMP.
- We investigate state-of-the-art hyper-heuristic techniques, many of them based on learning strategies, and critically compare their performance on the existing instances of the CPMP.
- Through thorough experimental analysis, we examine and discuss the impact of low-level heuristics.
- We compare the LNS and the best-performing hyper-heuristics to state-of-the-art methods for the CPMP on benchmark instances. Our approaches provide a lower average GAP value compared to the state of the art and in several cases better solutions. These computational results demonstrate the strength of LNS, and the robustness and potential of hyper-heuristics for the CPMP, as such methods may be employed in similar scenarios with minor adjustments.

This paper is organized as follows: in Section 2 we provide the problem definition. Then, in Section 3 we describe the LNS and its main components including a novel initialization heuristic. Subsequently, the proposed new low-level heuristics and the state-of-the-art hyper-heuristic techniques are explained in Section 4. Next, in Section 5 we present the experimental results and analyze the impact of low-level heuristics. Finally, we conclude our work and present ideas for future research in Section 6.

## Problem description

In order to define the CPMP, let’s consider a set of customers *I* with demand $q_{i}$ and with capacity values $Q_{i}$ for $i \in I$. Every customer *i* is a potential median. $d_{ij}$ represents the distance between customer *i* and customer *j*. If customer *j* is selected as median the decision variable $x_{jj} = 1$. If customer *i* is assigned to the median *j* then $x_{ij} = 1$ and 0 otherwise. The objective function value is the sum of the distances among customers and selected medians. The mathematical formulation of the CPMP according to Lorena and Senne (2004) is as follows:

1 $$\begin{aligned} z = min\sum _{\forall i\in I}\sum _{\forall j\in I}d_{ij}x_{ij} \end{aligned}$$

Subject to

2 $$\begin{aligned} \sum _{\forall j\in I}x_{jj}&= p \end{aligned}$$

3 $$\begin{aligned} \sum _{\forall j\in I}x_{ij}&= 1&\forall i \in I \end{aligned}$$

4 $$\begin{aligned} x_{ij}&\le x_{jj}&\forall i \in I,~\forall j \in I \end{aligned}$$

5 $$\begin{aligned} \sum _{\forall i\in I}q_{i}x_{ij}&\le Q_{j}x_{jj}&\forall j \in I \end{aligned}$$

6 $$\begin{aligned} x_{ij}&\in \{0, 1\}&\forall i \in I,~\forall j \in I \end{aligned}$$

Constraint (2) sets the number of selected medians to be *p*. Constraints (3) force that every customer is assigned to only one median. Constraints (4) prohibit assigning customers to closed medians. Constraints (5) ensure that the capacity values of the medians are not exceeded and the demand of every customer is satisfied. Constraints (6) give the domains of the decision variables.

## Large neighborhood search for the CPMP

This section presents the components of LNS for the CPMP, starting with the framework of LNS, followed by a novel initialization heuristic, destroy operators, and repair operator.

### Framework of LNS

The framework of LNS for CPMP is given in Algorithm 1. For the implementation of LNS, an initial solution is required. In this case, the initial solution contains a set of open medians and the assigned customers for each median. For a more efficient search process we declare a counter for each customer *i* that shows how many times this customer has been part of a sub-problem. This helps to prioritize other medians in the following steps of LNS. For the chosen median *j*, one of the available destroy operators $d_l\in \mathcal {D}$ is picked according to the roulette wheel selection approach. This sub-problem is passed to the repair stage *r*, where the MIP solver Gurobi is used to solve it. The new solution returned by Gurobi is accepted only if it is better (has a lower cost) than the current one. If that is the case, the solution is updated. This procedure is repeated until the time budget $t_{max}= 3600$ seconds is reached and the improved solution $s^*$ is returned. Such a scheme can help to find better solutions in every step of the algorithm by exploring large neighborhoods.

**Algorithm 1:**
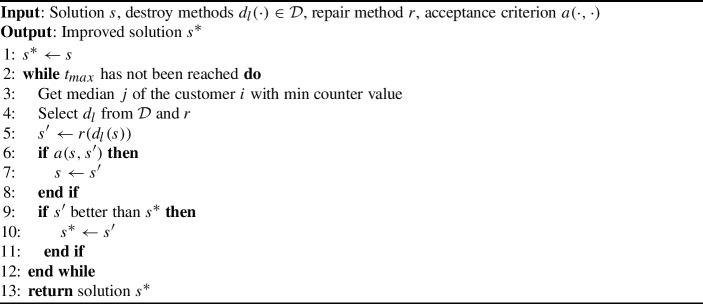
LNS for CPMP

### Initial solution

Both LNS and hyper-heuristics work by changing an existing solution, therefore, an initial solution needs to be obtained by an initialization heuristic. The basis for this was the heuristic by Mulvey and Beck (1984). It has two phases and starts from a random set of *p* medians, assigns all customers sorted by decreasing order of regret (distance between closest and second closest median) to the closest median where it fits according to capacity. Then it chooses the best median in each cluster and iterates the regret-based assignment until no further improvement larger than $\varepsilon$ is found. In the second phase all pairwise switches of customers are executed if they are feasible and improve the solution. The whole process is repeated multiple times, selecting the overall best solution. However, this heuristic does not scale well for the larger instances, especially due to the repeated quadratic check of pairwise customer exchanges and repeated regret computations. Therefore we introduce the following new heuristic: We repeat phase 1 for up to 10 times (at least 2, further iterations until a timeout of 10 seconds), while the second phase is replaced by only changing one median randomly from the best solution so far followed by the regret-based assignment again. This is repeated at least 10 times, further until 5 iterations without improvement and at most 60 seconds, even further only as long as consecutive improvements are found. The cutoff for improvements is set to $\varepsilon =1$.

**Algorithm 2:**
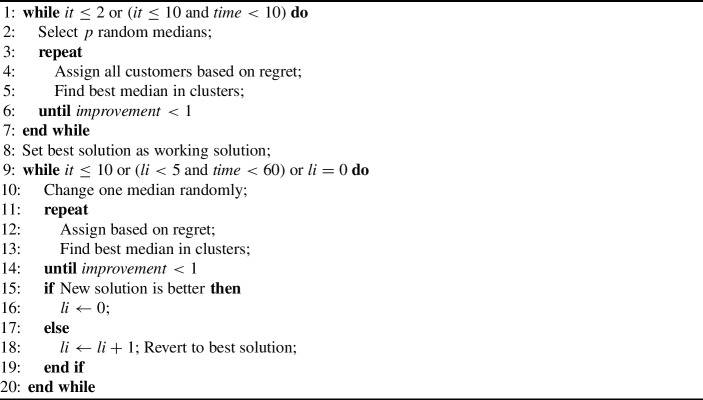
Initial heuristic

#### Usage of KD trees

Based on these changes, the largest contribution to runtime comes from the repeated assignment of customers based on the regret value. To compute the regret values, the difference between the distances to the closest and second closest median need to be computed for each customer. In a naive approach, this requires $|I|\cdot p$ distance computations, followed by sorting |*I*| arrays of size *p*. However, this can be sped up massively using kd-trees (Bentley 1975). While kd-trees have been used to compute closest medians (Gnägi and Baumann 2021), their use for regret computation is novel. Therefore, the assignment based on regret values is calculated as follows. A kd-tree for all medians is generated once, followed by |*I*| requests for the two closest medians (average complexity $\mathcal {O}(\log p)$). Their difference directly gives the regret value. For the assignment of each customer to a median, the two closest candidates are already known. Only when these are not possible based on the capacities, the distances to other medians are computed and sorted, but the number of occurrences is now expected to be much smaller than |*I*|. The evaluation section will show the tremendous speedup of our initial heuristic while obtaining better solutions than the previous heuristic. Note that the regret-based assignment sometimes fails due to customers with very high demand being assigned at a point where no median has enough remaining capacity. In this case the assignment is switched to decreasing order of demand.

### Destroy operators

In order to define the destroy operators employed in the algorithm, let’s consider the set of medians in the current solution. This set of medians contains all opened medians at that point. As for every customer in each instance the *x* and *y* coordinates are defined, we also use this information for the composition of operators. Given a selected median *m*, the operators are defined as below:

- Closest k medians in xy-plane (CKxy): For a selected median *m*, we determine its *k* closest medians in terms of Euclidean distance from the set of current opened medians in the *xy* plane.
- Closest k medians in x-axis (CKx): For a selected median *m*, we define its *k* closest medians in terms of Manhattan distance from the set of current opened medians based on *x* coordinates solely.
- Closest k medians in y-axis (CKy): For a selected median *m*, we define its *k* closest medians in terms of Manhattan distance from the set of current opened medians based on only *y* coordinates.

For the selected medians we identify their assigned customers. Then, the sub-problem in each iteration consists of these entities. Note that the sub-problems obtained by CKx and CKy are not subsets of the CKxy operator as they are based on different geometric shapes (elongated rectangles along the respective axis) that only partially overlap with circular selection of the CKxy operator.

### Repair operator

Initially, we experimented with Gurobi to solve the full problem using the model provided in Section 2. Based on our investigation, it appears that Gurobi can achieve optimal solutions for the majority of instances with up to 724 customers within a runtime of 3 hours. However, even for instances with less than 724 customers there are some cases that considerably increase the runtime of Gurobi. One component with high impact in the search process is the ratio of customers *n* to the *p* median value. For instances with a high *n*/*p* value, Gurobi needs a substantially greater amount of time to provide a solution. Selecting *p* medians and assigning customers to these medians seems to take long time in Gurobi as more customers should be allocated to each median. The possible selection of *p* medians is also dependent on the total capacity offered by the *p* medians compared to the total demand arising from customers. Based on our observations the dispersion of customers is also another feature that increases the hardness of an instance. These findings help for the configuration of parameters described in Section 5.2.

The mathematical model described in Section 2 is declared for the sub-problem chosen by the destroy operator and solved with Gurobi. In every step, we provide to Gurobi a warm start given by the current solution, which usually speeds up the search process.

## Hyper-heuristics for the CPMP

This section presents the main components of hyper-heuristics, a set of novel low-level heuristics, and an overview of the evaluated hyper-heuristics for the CPMP.

### Low-level heuristics

The proposed LLHs, which include destroy-and-repair, mutation, and improvement LLHs, are described below.

#### Destroy-and-repair

For this category of heuristics, we utilize the destroy operators part of the LNS procedure, combined with the same repair operator:

- Closest k medians in xy-plane (CKxy): For a certain median, select its *k* closest medians from the set of current open facilities based on Euclidean distance.
- Closest k medians in x-axis (CKx): For a certain median, select its *k* closest medians from the set of current open facilities based on Manhattan distance considering only *x* coordinates.
- Closest k medians in y-axis (CKy): Same concept based only on *y* coordinates.

Each of these operators is repaired with the MIP solver Gurobi similar as in the LNS. However, the size of the subproblem is used slightly differently (no tuning was performed for the hyper-heuristics): For instances with less than 750 customers, the number of customers in the subproblem is $50\%$ of the number total number of items *n*, and for the rest of the instances the number of items in each iteration is $\sim 500$. The time budget for each iteration of the Gurobi repair stage is set to be 100 seconds.

#### Mutations

Mutation heuristics allow changes that might yield worse objective function values:

- Random median change (RMC): Randomly select any of the currently open medians. Substitute this median with a randomly chosen customer from its assigned customers.
- Random customer change (RCC): Randomly select a customer and assign it to the nearest median with sufficient capacity.

#### Improvement heuristics

This group of heuristics results only in improved objective function values, otherwise the changes are discarded:

- Lowest close, highest open (LCHO): Identify the median $m_1$ with the lowest capacity used and its nearest median $m_2$. Identify the median with the highest capacity used $m_3$ and its $k-1$ closest medians $m_4..m_{k+3}$. Close one median in the first set $\{m_1, m_2\}$ and open a new median in the second set $\{m_3..m_{k+3}\}$. The capacity used $U_{j}$ for each median *j* would be the sum of the demands of their respective customers as in Equation (7).
  7 $$\begin{aligned} U_{j} = \sum _{\forall i\in I}q_{i}x_{ij} \quad \forall j \in I \end{aligned}$$
- Lowest close, random open (LCRO): Identify the median $m_1$ with the lowest capacity used and its nearest median $m_2$. Select randomly a median $m_3$ and find its $k-1$ closest medians $m_4..m_{k+3}$. Close one median in the first set $\{m_1, m_2 \}$ and open a new median in the set of all customers assigned to the open medians in the set $\{m_3..m_{k+3}\}$.
- Random close, random open (RCRO): Randomly pick two medians and identify for each its nearest median. Close one median from the set of medians with lower used capacity $\{m_1, m_2\}$ and open a new median from all customers assigned to the medians in the other set $\{m_3, m_4\}$.
- Rearrange closest k medians in xy-plane (RCKxy): For a selected median find its $k-1$ closest medians in terms of Euclidean distance. Substitute each median with any of its customers randomly and reassign customers based on regret values.
- Rearrange closest k medians in x-axis (RCKx): For a selected median find its $k-1$ closest medians based on *x* coordinates. Substitute each median with any of its randomly selected customers and reassign customers based on regret values.
- Rearrange closest k medians in y-axis (RCKy): Same concept based on *y* coordinates.
- Customer rearrangement (CR): Randomly select a median. Identify its closest median and rearrange their customers based on regret values.
- Max used capacity (MaxUC): Identify the median with the most used capacity and find its closest $k-1$ medians. Substitute each median with any of its randomly selected customers and reassign customers based on regret values.
- Min used capacity (MinUC): Identify the median with the least used capacity and find its closest $k-1$ medians. Substitute each median with any of its randomly selected customers and reassign customers based on regret values.
- Max cumulative distance (MaxCD): Identify the median with the highest sum of distances among its customers and find its closest $k-1$ medians. Substitute each median with any of its randomly selected customers and reassign customers based on regret values. The cumulative distance $Cum_{j}$ for any median *j* is defined in Equation (8).
  8 $$\begin{aligned} Cum_{j} = \sum _{\forall i\in I}d_{ij}x_{ij} \quad \forall j \in I \end{aligned}$$
- Min cumulative distance (MinCD): Identify the median with the lowest sum of distances among its customers and find its closest $k-1$ medians. Substitute each median with any of its randomly selected customers and reassign customers based on regret values.
- Check per cluster (CCl): For every cluster, check if any of its respective customers can be set as the new median for that cluster, if such setting results in a lower sum of distances among cluster members.
- Check per customer (CCu): Reassign each customer to the another median if the distance of the customer from the new median is smaller than then the distance from its previous median and if there is enough capacity to accommodate it. The order of customers is based on the descending distance of customers to their assigned median.
- Random selection (RS): Randomly select *k* medians from the set of open facilities. Substitute each median with any of its randomly selected customers and reassign customers based on regret values.

### Hyper-heuristics

We compare 17 different hyper-heuristics based on the HyFlex framework (Ochoa et al. 2012), which was introduced as part of the Cross-Domain Heuristic Search Challenge (Burke et al. 2011) for a uniform implementation of different hyper-heuristics.

- GIHH: The winner of CHeSC 2011 by Misir et al. (2011) uses an adaptive dynamic heuristic set to monitor the performance of each heuristic to select heuristics in different phases, finds effective pairs of heuristics, and uses a threshold accepting method. Despite its age and reports of several hyper-heuristics outperforming it on the CHeSC benchmark, it still provides very good results on many practical domains.
- L-GIHH (Lean GIHH): Designed to keep the power of GIHH while being less complex, this hyper-heuristic was obtained by performing Accidental Complexity Analysis on GIHH by Adriaensen and Nowé (2016), and was reported to provide similar or even slightly better performance compared to GIHH.
- HH-ALNS: Self-adaptive large neighborhood search by Laborie and Godard (2007) uses alternating perturbation and reconstruction moves, and learns weights for the LLHs based on their performance. Note that in this case the ALNS operates on LLHs, while in our proposed LNS we operate directly on the solution space and with fixed weights of operators.
- SW-ALNS: Sliding window ALNS extends HH-ALNS with time windows based on Thomas and Schaus (2018). The time windows take the different time taken by various LLHs into account when calculating the weights.
- BSW-ALNS: Bigram sliding window ALNS uses the time windows, and additionally the weights for reconstruction moves are not learned independently, but based on the preceding perturbation move.
- CH-UN, CH-BI, CH-FR, CH-PR: Chuang (2020) introduces a range of methods based on the concept of solution chains in his thesis. If any solution in the chain is better than the starting solution, it is accepted and the chain stopped, otherwise the solution is reverted to the start of the chain. The lengths of the chains are chosen according to the Luby sequence, which uses short sequences frequently, and exponentially longer sequences rarer. In the uniform version (CH-UN) the LLHs are chosen randomly with a uniform distribution. For CH-BI (Bigram), probabilities are set according to the previous heuristic in the chain and the number of times this pair of heuristics occurred in successful chains. In CH-FR (Frequency), the probability to choose a heuristic depends on the number of times it appeared in successful chains. Finally, CH-PR (Pruning) uses a warm-up period to determine bad heuristics that are pruned, and random uniform selection afterwards.
- HAHA: The hyper-heuristic by Lehrbaum and Musliu (2012) switches between working on a single solution and a pool of solutions, and applies an adaptive strategy for selecting LLHs.
- FS-ILS: Fair-Share Iterated Local Search by Adriaensen et al. (2014) uses iterated local search with a conservative restart condition. Perturbations are picked according to their previous acceptance rate by using a variant of the Metropolis condition.
- TS-ILS: This hyper-heuristic by Adubi et al. (2021) is based on iterated local search. It applies a probabilistic learning technique (Thompson Sampling) to configure the behaviour of the ILS and reported very good results on the HyFlex domains.
- EA-ILS: Based on FS-ILS, this hyper-heuristic by Adubi et al. (2022) uses an evolutionary algorithm to control the selection of perturbative LLHs, and was also reported to provide very good results on the HyFlex domains.
- Q-LEARNING, SARSA, E-SARSA: This set of methods by Mischek and Musliu (2022) uses reinforcement learning to learn the next LLH to apply, together with solution chains. The different versions use different update rules, which are Q-learning, SARSA, and expected SARSA.
- LAST-RL: This hyper-heuristic by Kletzander and Musliu (2023) also uses reinforcement learning, but with a more complex state representation and exploration probabilities based on iterated local search, and reports high-quality results on several real-life scheduling domains.

## Experimental evaluation

The experiments were run on a computing cluster, equipped with two Intel Xeon E5-2650v4 @ 2.20 CPUs with 12 cores and on a single thread, using up to 25.6 GB of RAM. Gurobi v11.0.0 was used in the experiments. The proposed LNS was implemented in Python and run using PyPy 7.3.11, Python 3.8.16. With a timeout of one hour, 10 runs per instance were performed. The tested hyper-heuristics were implemented in the Java HyFlex framework (Ochoa et al. 2012). The execution was done with OpenJDK 17.0.3 while the low-level heuristics were implemented in Python and run using the same setup as LNS. The maximum runtime is one hour (combined execution time of Python and Java excluding time for communication between the processes) and 10 runs are conducted for each method, for each instance.

To test the proposed LNS and hyper-heuristics we use four distinct data sets available in the literature^1^$$^,$$^2^. The size of every instance is denoted as $$n \times p$$ where *n* is the number of customers and *p* is the number of the determined medians to be opened. The first data set was introduced by Lorena et al. (2003) and has five instances labeled as p$$3038\_600$$ (size $$3038 \times 600$$) to p$$3038\_1000$$ (size $$3038 \times 1000$$). The second data set was presented by Lorena and Senne (2004) and contains real-life instances. These six instances that follow the naming sjc1 to sjc4*b* have a size from $$100 \times 10$$ to $$402 \times 40$$. The third data set was introduced by Stefanello et al. (2015) and includes 30 instances with the smallest size being $$318 \times 5$$ and the largest one being $$4461 \times 1000$$. The last dataset provided by Gnägi and Baumann (2021) includes very high dimensional instances with at most $$498\,378$$ customers and up to $$2\,000$$ medians.

We compare our results from LNS and from the most effective hyper-heuristics for these data sets with the state-of-the-art approaches including a matheuristic proposed by Stefanello et al. (2015) named as IRMA, and the matheuristic introduced by Gnägi and Baumann (2021) denoted as GB. As the implementation of IRMA is not publicly available we utilize MOPS (Millions of Operations Per Second) for a fair comparison. First we obtain the MOPS for the hardware and then we provide adjusted runtimes of this method’s hardware with our hardware specifications. The GB approach is publicly available, making it possible to run it in the same environment as LNS and hyper-heuristics. For comparison, we use the GAP value defined as follows:

9 $$\begin{aligned} \textrm{GAP}_{\textrm{sol}} = \frac{(Z_{sol}-\textrm{BKS})}{\textrm{BKS}}\cdot 100 \end{aligned}$$

In Equation (9), $Z_{sol}$ stands for the objective function value of the solution, and BKS represents the best-known solution reported in the literature. Note that the best-known solutions for the first 3 datasets were reported by Stefanello et al. (2015), who state that these are best results obtained from different configurations they tried. Additionally, the best-known solutions for the last dataset are provided by Gnägi and Baumann (2021). In the following subsections, our analysis is based on the first 3 datasets, unless indicated otherwise.

### Initial solution results

**Table 1:** Comparing versions of the initialization heuristic

Instance subset	Original		New		New + kd-trees	
	GAP	time	GAP	time	GAP	time
sjc	6.4	4.4	**4**.**5**	**0**.**6**	4.8	0.9
lin318	13.7	5.9	10.6	1.3	**9**.**6**	**1**.**2**
ali535	15.6	13.9	**13**.**0**	3.5	14.0	**1**.**7**
u724	10.5	24.6	**8**.**4**	7.4	8.7	**2**.**6**
rl1304	14.2	66.6	**11**.**6**	20.3	**11**.**6**	**5**.**3**
pr2392	10.8	191.2	10.5	40.5	**9**.**0**	**11**.**0**
fnl4461	9.1	610.7	9.3	129.3	**7**.**6**	**29**.**4**
p3038	23.2	743.8	24.7	152.1	**22**.**8**	**22**.**4**
All	12.8	202.7	11.4	43.3	**10**.**9**	**9**.**1**

Bold represent the best solution

Table 1 shows the evaluation of the new initialization heuristic. It compares the original method by Mulvey and Beck (1984), our new method with naive regret computation, and our new method using kd-trees for the regret computation (5 runs each for each instance). For each method it shows the average GAP and the average runtime in seconds for each subset of instances (each containing 5 to 6 instances).

The results clearly show the dominance of our new method. The average GAP drops slightly, while the average runtime drops by 78.6 % using the new method without kd-trees and by impressive 95.5 % using kd-trees. Especially for the two largest categories, fnl4461 and p3038, the runtime drops by more than an order of magnitude while the average results are better than before. As expected, the new method with and without kd-trees provides very similar gaps except for those larger instances where without kd-trees the timeout is hit. Note that kd-trees are beneficial even for smaller instances, with only the sjc set showing a slighly lower runtime without kd-trees. This initialization heuristic has been used for the LNS and for all the hyper-heuristics.

### Parameter configuration for LNS

The parameters of the LNS are the repair stage time, the number of customers involved in each iteration, and the weights of the proposed operators. The first two parameters, illustrated in Table 2, are manually set while the weights of the operators are set by means of the automatic parameter configuration tool irace (López-Ibáñez et al. 2016). We determined the number of customers to be included in every iteration based on the number of customers *n*. We set this value to $75\%$ of the number of customers for instances with less than 450 customers, to $50\%$ of the number of customers for instances with less than 750 customers and 500 for other instances. The time given to Gurobi is 150 seconds and a warm start is provided in each step to help the search process. For instances with a high number of medians, we consider each customer in the iteration as a candidate median. On the other hand, for instances with a high ratio of *n*/*p* we narrow down the list of candidate medians to only the 30 nearest customers from every median. For example, in an iteration where there are 2 free medians and 500 customers, Gurobi does not find a feasible solution even if we let it run for 1 hour. Hence, for such instances we restrict the search to 30 nearest customers per median.

A crucial element of the LNS is the selection of the operators which in our implementation is based on the roulette wheel principle. At each iteration, a random number between 0 and 1 is generated and the operator is selected according to its respective weight. To determine the weights of the operators, we use the automatic parameter configuration tool irace (López-Ibáñez et al. 2016). The instance tuning set consists of 11 instances and a total budget time of $$349\,200$$ seconds has been used. The weights $$w_{l}$$ of operator *l* for $$ l = 1, 2, 3$$ as resulted from irace are: $$w_{1} = 0.30$$, $$w_{2} = 0.35$$, $$w_{3} = 0.35$$.

**Table 2:** Parameter values used for the LNS

Parameter	Value	Condition
Repair stage time	150 seconds	-
Number of customers in each iteration	500	($n > 750$)
	$0.50 \cdot n$	($450< n <= 750$)
	$$0.75 \cdot n$$	($$ n <= 450$$)

### Ablation analysis for LNS

**Fig. 1:**
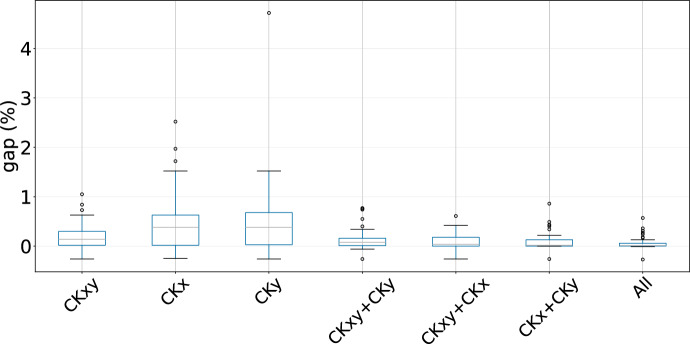
Comparison of operators’ performance

To fully examine the efficiency of each operator, we also conduct an ablation analysis, where we utilize only one of the operators separately, combinations of them two by two, and all of them. We perform 10 runs with different seeds in each of these cases: CKxy, CKx, CKy, CKxy $+$ CKy, CKxy $+$ CKx, CKx $+$ CKy, and CKxy $+$ CKx $+$ CKy. From Figure 1, we can say that using any of the three operators alone is not a good idea. Especially in the case of using only CKx, or only operator CKy some average GAP values (as in Equation 9) higher than $1 \%$ are obtained. On the other hand, using a combination of two operators seems to have better results. However, apart from yielding an average GAP value higher than the combination of using the all operators together, only 2 new best solutions are found by using operator CKxy and CKy, while combining operator CKxy and CKx or CKx and CKy found more new solutions, yet not as good as employing all the operators. Hence, we can conclude that the use of 3 operators together is more efficient and brings value in discovering better solutions and providing a lower average GAP value. Such claim is also confirmed by irace, as it recommended to use each operator with these weights: $w_{1} = 0.30$, $w_{2} = 0.35$, $w_{3} = 0.35$.

### Comparison of hyper-heuristics

**Fig. 2:**
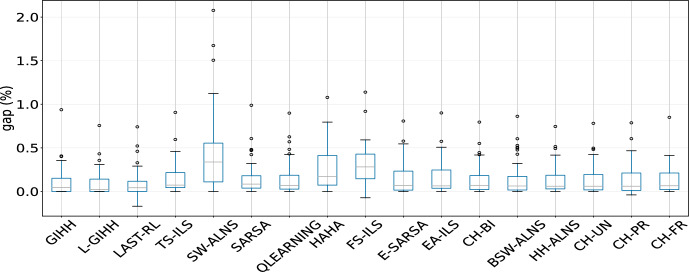
Comparison of hyper-heuristics’ performance based on average GAP values

In Figure 2 we provide the average GAP values for all the evaluated hyper-heuristics. The best performing hyper-heuristic is L-GIHH, followed by GIHH, and LAST-RL, with all average GAP values smaller than 1. Furthermore, LAST-RL provides several negative GAP values that indicate new better solutions than the existing ones are found. Other hyper-heuristics BSW-ALNS, CH-UN, CH-PR, CH-FR, CH-BI, HH-ALNS, QLEARNING, SARSA, E-SARSA, TS-ILS, EA-ILS have a similar performance. Such results demonstrate that our novel set of LLHs is robust in providing high quality solutions with different hyper-heuristics.

### Comparison to the state of the art

In this subsection we compare LNS and the three best performing hyper-heuristics L-GIHH, GIHH, and LAST-RL with the state-of-the-art approach GB by Gnägi and Baumann (2021). In GB (Gnägi and Baumann 2021), they apply a global optimization stage for obtaining an initial solution while their local optimization stage at the start gives priority to medians that do not fully use their available capacity. For a full comparison, we use GB with the parameters declared in Gnägi and Baumann (2021), and we also tune the parameters of GB using irace (López-Ibáñez et al. 2016). In the tuning process, we use the same training instances and budget time as for LNS. In the reported results, GB stands for the algorithm with the declared parameters as in Gnägi and Baumann (2021) and GB$_{tuned}$ represents the GB algorithm with the recommended parameters from irace.

**Fig. 3:**
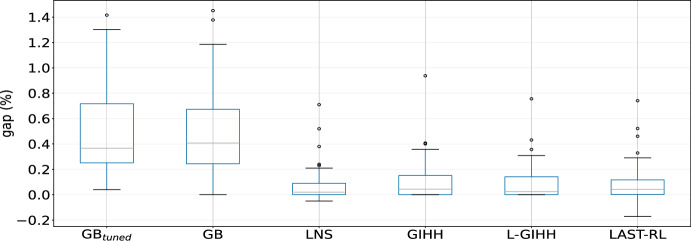
Comparison of GB$_{tuned}$, GB, LNS, GIHH, L-GIHH, LAST-RL

GB$_{tuned}$, GB, LNS, and hyper-heuristics have undergone 10 runs with a time limit of 3600 seconds. For a more detailed comparison regarding all datasets except the huge instances (Gnägi and Baumann 2021), the boxplots of GB$_{tuned}$, GB, LNS, GIHH, L-GIHH, and LAST-RL (for the average GAP values over 10 runs) are given in Figure 3. Both GB$_{tuned}$ and GB produce some outliers (outside the visualized range in Figure 3), as some instances spend most of the time limit in the global optimization phase. LNS has an average GAP of 0.08, GIHH, L-GIHH, and LAST-RL have an average GAP value of 0.10, GB a value of 7.23, and GB$_{tuned}$ a value of 7.06. Therefore, LNS, GIHH, L-GIHH, and LAST-RL outperform both GB$_{tuned}$ and GB.

**Fig. 4:**
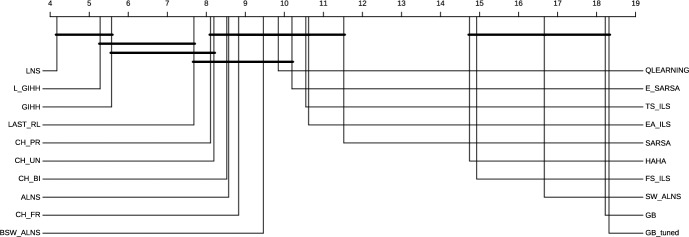
Critical difference plot for all instances of the first 3 datasets

We have also performed statistical tests using the R script scmamp (Calvo and Santafé Rodrigo 2016). The Friedman test reports a p-value smaller than $2.2 \times 10^{-16}$ for the results of all hyper-heuristics, LNS, GB, and GB$_{tuned}$. Thus, not all of them perform the same. Additionally, we check the Nemenyi post-hoc test. These results are displayed with a critical difference plot in Figure 4. Algorithms are ranked based on their performance for all instances where lower values show better performance. Algorithms joined by a bold horizontal bar have the same performance statistically. From these results, we can say that LNS performs better than L-GIHH, GIHH but not with statistical significance. LNS, L-GIHH, GIHH, and LAST-RL perform better than GB$_{tuned}$ and GB with significant difference. The minimum, average, and maximum values of GB, GB$_{tuned}$, LNS, L-GIHH, GIHH, and LAST-RL are reported in the Appendix A. Furthermore, our approaches also find new best solutions, which are presented in the Appendix, Table 7.

**Table 3:** Comparison of GB$_{tuned}$, GB, LNS, GIHH, L-GIHH, and LAST-RL based on average GAP values (%)

Instance	GB$_{tuned}$	GB	LNS	GIHH	L-GIHH	LAST-RL
XMC10150_100	0.18	0.27	$-$0.56	$-$0.84	$-$**0**.**92**	-0.84
XMC10150_1000	0.46	**0**.**22**	7.06	1.31	1.06	4.36
XMC10150_2000	7.25	5.16	18.22	2.77	**2**.**36**	9.44

Bold represent the best solution

Another tested dataset is the one by Gnägi and Baumann (2021) composed of very large instances. Our methods do not scale well on these instances, as most instances run into a memory error (using more than 20 GB of RAM). However, their size is unrealistic for typical applications of the CPMP, and GB uses up to 128 GB of RAM in their evaluation. We show the results obtained by GB$_{tuned}$, GB, LNS, GIHH, L-GIHH, and LAST-RL for the instances XMC10150 (with $10\,150$ customers) from this dataset in Table 3. For the XMC10150_100 instance, LNS, GIHH, L-GIHH, and LAST-RL manage to find new better solutions compared to GB and GB$_{tuned}$. For the instance XMC10150_1000, GB and GB$_{tuned}$ perform better than our approaches, and for the instance XMC10150_2000, L-GIHH provides the lowest average GAP value. For the remaining instances from this dataset, the GB approach (Gnägi and Baumann 2021) represents the state of the art.

#### Comparison to IRMA

**Fig. 5:**
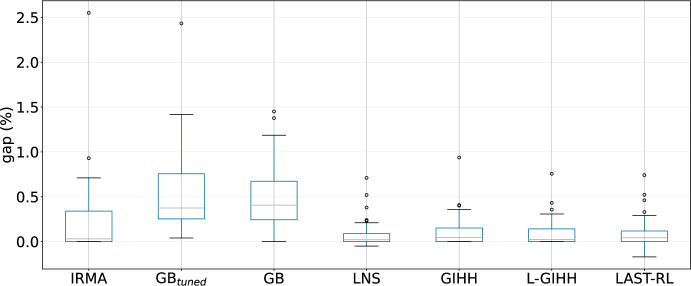
Comparison of IRMA, GB$_{tuned}$, GB, LNS, GIHH, L-GIHH, LAST-RL

The comparison with the IRMA approach by Stefanello et al. (2015) is concluded in a separate section accounting for differences in solvers and use of normalized runtime. In IRMA (Stefanello et al. 2015) the primal heuristic proposed by Mulvey and Beck (1984) is used by applying uniform customer assignments rather then regret-based customer assignment. Their methodology uses these steps: removing variables based on demand and on neighborhood definitions, using an exact solver for the reduced model, and passing the results of the solver to a post-optimization stage. They use timeouts only for particular phases, and stop when reaching a local optimum, which takes up to one hour. The average GAP values for IRMA, GB$_{tuned}$, GB, LNS, GIHH, L-GIHH, LAST-RL are presented in Figure 5 (some outliers of GB$_{tuned}$ and GB not visible). Again, our methods offer a lower average GAP value compared to IRMA.

### Solution quality over time

**Fig. 6:**
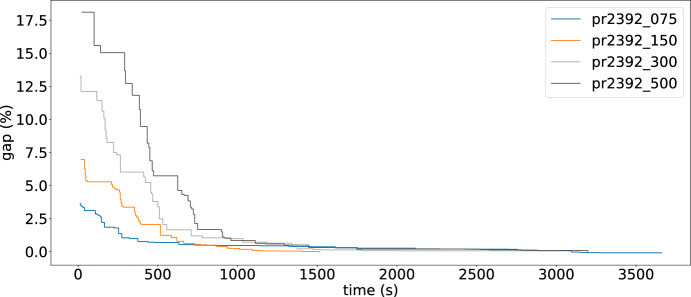
GAP (%) values over time for LNS

**Fig. 7:**
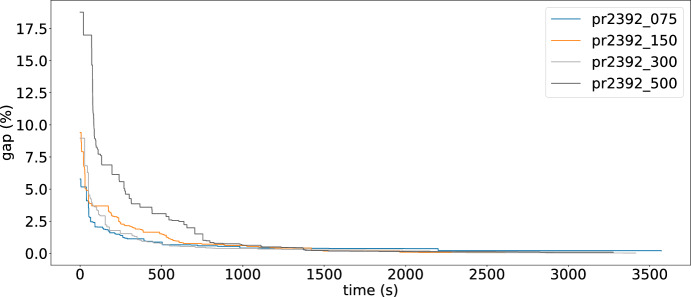
GAP (%) values over time for L-GIHH

To illustrate the change of the cost values over time, we select 4 instances with the same number of customers and varying *p* value. For these instances, the changes in GAP values by the LNS approach and by L-GIHH hyper-heuristic are presented in Figure 6 and in Figure 7 respectively. LNS exhibits larger improvements across iterations whereas L-GIHH shows gradual improvements in the GAP values. However, for both these methods, all instances reach a GAP value of almost 0 approximately halfway through the time budget.

### Analysis of LLHs

As the designed LLHs are an essential part of the hyper-heuristics, in this section we investigate their contribution to the performance of the evaluated techniques. We conduct 10 runs with different seeds for the three best performing hyper-heuristics in the following cases: removing destroy-and-repair LLHs (DR), removing mutation LLHs (MU), removing improvement LLHs that use regret values for the assignment of customers (RB), removing improvement LLHs that check all possible changes (GI), and removing both mutation and improvement LLHs (MI).

**Fig. 8:**
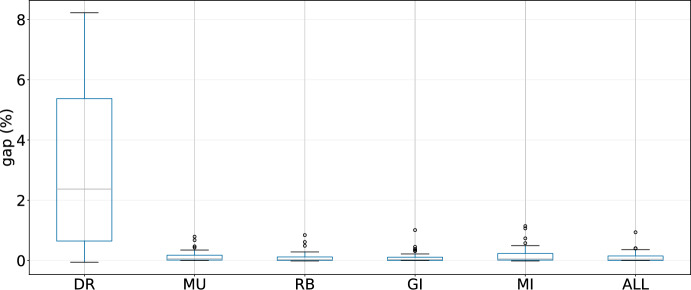
Results of GIHH for different scenarios

**Fig. 9:**
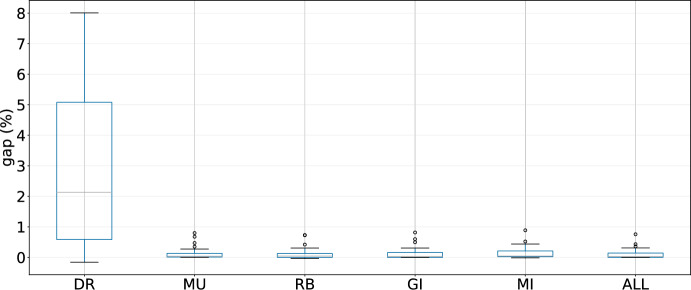
Results of L-GIHH for different scenarios

**Fig. 10:**
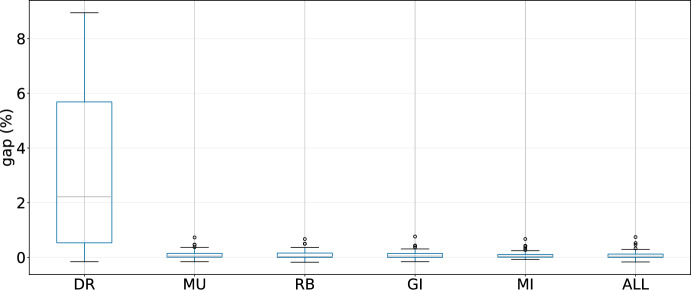
Results of LAST-RL for different scenarios

For these experiments, the results for GIHH are shown in Figure 8, for L-GIHH in Figure 9, and for LAST-RL in Figure 10. The last boxplot for each hyper-heuristic (shown as ALL in the respective figure) displays the results when all LLHs are active. The pattern is clear, when the destroy and repair LLHs (DR) are removed for GIHH, L-GIHH, and LAST-RL, a substantial increase in the average GAP values is noticed which indicates a great importance of these LLHs in the performance of the hyper-heuristics. In other cases (MU, RB, GI, MI), the average GAP values increase less than in the previous case, but now there are more outliers in terms of average GAP values. Also, the hyper-heuristics fail to find as many new best-known solutions as in the configuration with all LLHs. These findings confirm the adequacy of the proposed and tested LLHs for a good performance of the hyper-heuristics.

### Usage of LLHs

Finally, we evaluated how LLHs were used by the three best hyper-heuristics in detail. This includes their runtimes, the number of LLHs calls as well as the relative frequency of different LLHs for the different hyper-heuristics as well as on different sets of instances.

#### Runtime

The runtime of the LLHs varies considerably as the ruin-and-recreate LLHs are much slower than the others. These three LLHs have runtimes of around 0.14 to 4.4 seconds for the sjc instances as well as instances 1, 2, and 4 from the lin318 instances. Runtimes for the other instances can raise considerably, with average values of up to 86 seconds with the highest values typically achieved by the first instances of the pr2392 and fnl4461 subsets, as these have the highest number of customers per median, and therefore even with the adaptive selection of the subproblem size, the subproblem is not easy to solve.

The mutations and local search heuristics are very fast, with random median change RMC being the fastest in less than 0.01 seconds, and all mutations and local search LLHs have average runtimes of only up to 0.39 seconds over all instances. Even for the larger instances, runtimes rarely exceed 1 second, with the first instance of set fnl4461 being the only notable exception with runtimes of up to 2 seconds.

#### LLH calls

The number of LLH calls varies significantly depending on both the instance and the strategy of the hyper-heuristic, as few calls to ruin-and-recreate LLHs take as much time as a large number of other LLH calls. In general, LAST-RL prefers the slow ruin-and-recreate heuristics, leading to much lower numbers of LLH calls. In the available hour of runtime, it makes between around 400 and up to more than $100\,000$ calls, while GIHH and L-GIHH, which prefer the faster LLHs, make around 1400 up to more than $300\,000$ calls.

#### Relative LLH frequencies

**Fig. 11:**
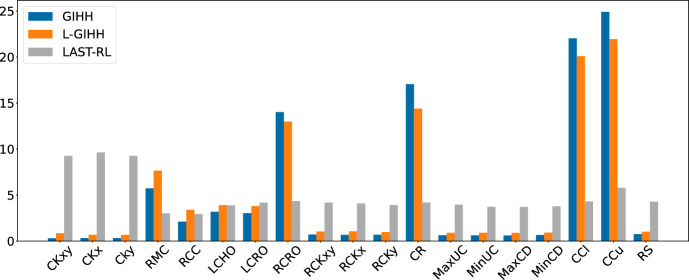
Average LLH frequencies

Figure 11 shows the average frequencies of the individual low-level heuristics for the 3 best hyper-heuristics. It is interesting to see the very different preferences of the hyper-heuristics. First, LAST-RL clearly prefers the ruin-and-recreate LLHs, giving each of them around 10 percent of all calls. GIHH and L-GIHH, on the other hand, use these LLHs much more sparsely, especially GIHH with values of around 0.3 percent. Note that this only translates to LAST-RL making around twice as many calls to ruin-and-recreate, since the other heuristics are that much faster, GIHH and L-GIHH can pack many more calls into the extra runtime that LAST-RL spends on ruin-and-recreate. Regarding mutations, GIHH and L-GIHH favor RMC, while LAST-RL uses them almost equally. Regarding local search, GIHH and L-GIHH have clear favorites, with the winners being check per customer (CCu) before check per cluster (CCl), customer rearrangement (CR), and random close, random open (RCRO). While LAST-RL uses these LLHs more equally, CCu also sticks out as the winner in this case.

**Fig. 12:**
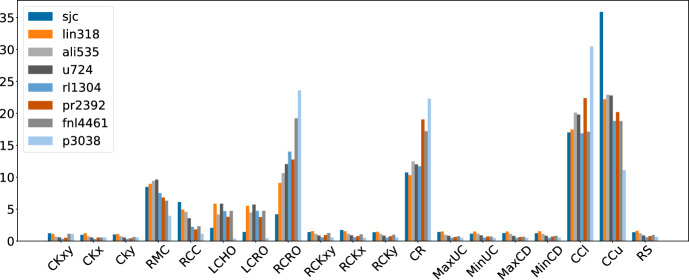
Average LLH frequencies for different instance subsets (L-GIHH)

While the overall selection frequencies are already interesting, a more detailed analysis reveals even more patterns. Figure 12 shows the frequencies used by L-GIHH for the different subsets of instances. While the general trends persist for all subgroups, several interesting conclusions can be drawn. In general, the larger the number of customers, the lower the preference for CCu gets, while the preferences for RCRO, CR, and CCl raise. CCl, RCRO, and CR are especially preferred for p3038.

The picture is very similar for GIHH, while LAST-RL in particular shows increased use of ruin-and-recreate for fnl4461 and p3038 compared to all other subsets.

**Fig. 13:**
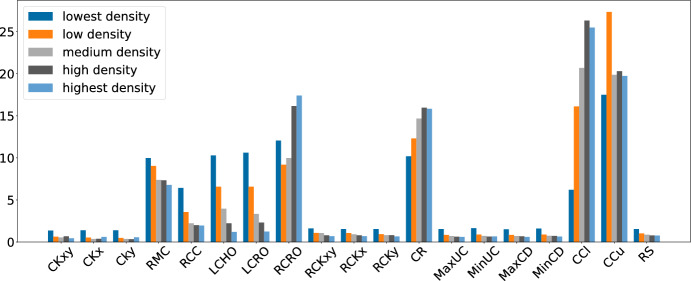
Average LLH frequencies for different instance densities (L-GIHH)

Figure 13 shows interesting patterns in a different direction, which is instance density. For each of the subsets lin318, ali535, u724, rl1304, pr2392, and fnl4461, the five instances have increasing density of medians. Figure 13 groups the instances by rising density, allowing to see a clear trend: For low density, the mutations as well as the local search heuristics that simultaneously close and open medians (LCHO, LCRO, RCRO) are used much more often, while the most used LLH (CCu) is again used frequently as in the overall average. Further, ruin-and-recreate is used slightly more regularly, even though still rarely. For increasing density, there is a clear trend to reduce the use of mutations RMC, RCC along with LCHO, LCRO, and increase the use of the most frequent LLHs. An exception is CCu, which has constant high usage with a large outlier in the second group. Again, the picture is very similar for GIHH (including the outlier).

**Fig. 14:**
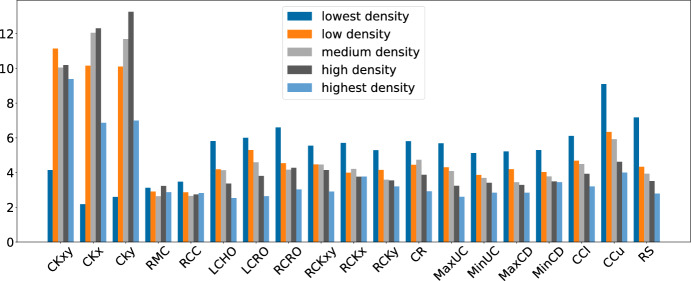
Average LLH frequencies for different instance densities (LAST-RL)

Finally, Figure 14 shows the same analysis for LAST-RL. While it is harder to see an overall trend, it is clear that the smallest density leads to a very different picture than the other densities, with greatly reduced use of ruin-and-recreate, and higher use of most of the local search heuristics.

## Conclusions

In this paper, we presented an LNS approach and a study of hyper-heuristics for the CPMP, where both LNS and the best hyper-heuristics perform better with statistical significance than the previous state of the art for instances with less than 5000 customers. Within the framework of LNS, we introduced new destroy operators that provide better results for some instances. Specifically, we define the sub-problem selecting the closest open facilities based on both x and y coordinates, based on only x coordinate, or based solely on y coordinate. For the selection of an initial facility we provide a selection mechanism based on the appearance of customers in each iteration. Considering the employed operators, we experimented with the automatic parameter configuration tool irace and we use its results in our proposed algorithm. Taking into account the power of MIP solvers, we utilize Gurobi in the repair stage of the LNS. Additionally, we demonstrated the effectiveness of a high-level, problem-independent approach based on hyper-heuristics, showing its capability to yield competitive results and, in some cases, even provide better results than problem-specific methods. Previous methods for the CPMP, a highly relevant practical problem, often incorporate advanced solving mechanisms and hybrid methods specifically tailored to address this problem. While hyper-heuristic methods necessitate good low-level heuristics or search operators, the advanced search mechanism that iteratively selects one of the operators in each iteration is general and independent of the problem domain. Our work demonstrated the robustness of several state-of-the-art hyper-heuristic techniques, consistently delivering very good results for the CPMP. Our experiments also underscored the importance of the novel low-level heuristics provided for this problem domain. The identified low-level heuristics, especially the combination of ruin-and-recreate LLHs with local search heuristics such as check per customer and cluster (CCu, CCl), customer reassignment (CR), and random close, random open (RCRO), were found to be crucial for achieving very good results. Our future plans include generating and evaluating instances with even more diverse structures, and applying the proposed approaches to related problems domains like uncapacitated p-median or capacitated clustering.

## Detailed results

Table 4, 5, 6, 7

**Table 4:** Detailed results for GB$_{tuned}$ and GB. The first column lists the instances, and the second column reports the best-known solutions. Columns 3–5 show the minimum, average, and maximum values for GB$_{tuned}$, while the last three columns report the same statistics for GB

instance	bks	GB$_{tuned}$			GB		
		min	avg	max	min	avg	max
lin318_005	180281.20	180615.03	181744.98	183543.00	180422.49	181151.87	181992.41
lin318_015	88901.56	88901.56	89004.80	89113.70	88911.60	88976.25	89105.74
lin318_040	47988.38	47988.38	48007.52	48046.39	47988.38	47988.38	47988.38
lin318_070	32198.64	32198.64	32235.15	32330.24	32198.64	32233.36	32302.40
lin318_100	22942.69	22942.69	22956.91	22994.39	22942.69	22960.74	22983.34
ali535_005	9956.77	9963.43	9992.66	10008.54	9958.50	9986.61	10008.54
ali535_025	3695.15	3695.35	3718.76	3755.18	3697.30	3723.72	3753.39
ali535_050	2461.41	2467.60	2521.28	2850.62	2460.93	2652.94	4042.56
ali535_100	1438.42	1725.52	3035.63	3781.11	2141.91	2882.74	3471.75
ali535_150	1032.28	1695.21	2654.89	2962.60	2025.49	2782.52	3169.50
u724_010	181782.96	182285.14	183221.48	184253.16	182418.89	182952.75	183665.88
u724_030	95034.01	95145.21	95605.90	96167.41	95222.27	95485.92	95667.02
u724_075	54735.05	54886.33	54994.30	55227.91	54860.08	54976.11	55157.07
u724_125	38976.76	39002.93	39064.09	39188.29	39010.74	39066.86	39192.14
u724_200	28079.97	28085.93	28120.50	28176.59	28087.68	28138.31	28182.15
rl1304_010	2146484.10	2153576.64	2166387.46	2188098.64	2148865.43	2170277.53	2190765.04
rl1304_050	802283.41	805358.18	809675.49	815029.90	803527.94	809986.70	814926.87
rl1304_100	498090.74	498683.49	500280.34	502041.66	499855.40	501006.68	503390.41
rl1304_200	276977.60	277385.47	277904.25	278597.50	277431.03	277761.14	278300.70
rl1304_300	191224.85	191407.13	191660.74	192055.69	191368.61	191632.55	192314.17
pr2392_020	2235376.73	2239257.22	2254170.74	2270358.24	2236915.51	2253187.49	2286429.30
pr2392_075	1092294.02	1096638.46	1099961.26	1104350.62	1095615.21	1101318.28	1107062.41
pr2392_150	711111.25	715100.44	715846.46	717328.04	714591.47	716092.56	717337.96
pr2392_300	458145.29	459353.80	459755.02	460218.42	459241.94	459679.99	460011.99
pr2392_500	316042.97	316348.87	316872.09	317290.07	316383.49	317015.17	317497.86
fnl4461_0020	1283536.73	1293126.18	1300259.62	1310404.65	1294807.73	1301215.98	1306904.40
fnl4461_0100	548909.01	555089.17	556680.56	559588.84	554280.55	556872.96	560748.19
fnl4461_0250	335888.87	338040.32	339188.86	340064.84	339324.60	339871.30	340451.77
fnl4461_0500	224662.49	225963.95	226284.57	226859.51	225841.73	226196.39	226556.89
fnl4461_1000	145862.38	146222.95	146407.56	146652.34	146275.61	146495.84	146733.61
p3038_600	122711.17	123189.69	123429.65	124054.86	123110.64	123263.40	123384.62
p3038_700	109677.30	109863.02	110041.19	110179.83	109892.46	110193.95	110746.38
p3038_800	100064.94	100581.35	101031.76	102179.42	100337.89	100702.22	101291.25
p3038_900	92310.09	92442.78	92670.44	92837.73	92509.69	92594.71	92739.56
p3038_1000	85854.05	86051.46	86148.45	86420.17	86056.81	86177.87	86468.16
sjc1	17288.99	17288.99	17323.32	17385.23	17288.99	17298.62	17385.23
sjc2	33270.94	33270.94	33363.73	33505.34	33346.52	33374.25	33505.34
sjc3a	45335.16	45335.16	45377.00	45444.88	45335.16	45374.41	45430.81
sjc3b	40635.90	40635.90	40739.46	40868.55	40635.90	40766.92	40873.16
sjc4a	61925.51	61925.51	62080.59	62176.98	61925.51	62062.51	62176.98
sjc4b	52458.02	52499.46	52612.57	52733.20	52495.60	52657.06	52848.29

**Table 5:** Detailed results for LNS and L-GIHH. The first column lists the instances, and the second column reports the best-known solutions. Columns 3–5 show the minimum, average, and maximum values for LNS, while the last three columns report the same statistics for L-GIHH

instance	bks	LNS			L−GIHH		
		min	avg	max	min	avg	max
lin318_005	180281.20	180281.21	180281.21	180281.21	180281.21	180282.26	180282.38
lin318_015	88901.56	88901.56	88901.56	88901.56	88901.56	88901.56	88901.56
lin318_040	47988.38	47988.37	47988.38	47988.38	47988.38	47988.38	47988.38
lin318_070	32198.64	32198.63	32198.64	32198.64	32198.64	32198.64	32198.64
lin318_100	22942.69	22942.69	22942.69	22942.69	22942.69	22942.69	22942.69
ali535_005	9956.77	9956.77	9956.77	9956.77	9956.77	9956.77	9956.77
ali535_025	3695.15	3695.15	3699.07	3717.15	3695.15	3701.11	3717.58
ali535_050	2461.41	2461.06	2467.41	2478.80	2460.81	2465.61	2477.94
ali535_100	1438.42	1434.08	1441.15	1447.10	1437.62	1440.46	1446.57
ali535_150	1032.28	1030.15	1033.91	1037.44	1029.46	1032.42	1034.47
u724_010	181782.96	181783.70	182197.54	182572.83	181776.99	182128.49	182562.16
u724_030	95034.01	95034.01	95080.49	95112.84	95034.01	95073.72	95112.84
u724_075	54735.05	54735.05	54743.33	54776.47	54735.05	54735.05	54735.05
u724_125	38976.76	38976.76	38983.60	38999.56	38976.77	38976.77	38976.77
u724_200	28079.97	28079.97	28080.08	28081.11	28079.97	28079.97	28079.97
rl1304_010	2146484.10	2146679.91	2154713.06	2172141.29	2147766.32	2155748.25	2172073.13
rl1304_050	802283.41	802280.47	803754.22	811074.42	802280.47	804755.56	810170.59
rl1304_100	498090.74	498090.73	498156.33	498354.76	498090.73	498161.06	498340.39
rl1304_200	276977.60	276977.59	277046.34	277242.07	276977.61	277038.19	277287.99
rl1304_300	191224.85	191224.84	191242.60	191275.89	191224.84	191240.16	191275.90
pr2392_020	2235376.73	2229894.92	2235433.85	2249593.27	2229588.62	2237688.99	2246955.59
pr2392_075	1092294.02	1090120.88	1091763.79	1094170.51	1090361.67	1092588.43	1097143.85
pr2392_150	711111.25	711039.65	711208.62	711649.26	711093.89	711513.32	712561.40
pr2392_300	458145.29	458170.02	458267.54	458438.29	458190.04	458376.23	458658.07
pr2392_500	316042.97	316069.76	316255.58	316495.88	316099.96	316472.95	317012.66
fnl4461_0020	1283536.73	1285088.01	1290181.99	1298402.49	1285368.04	1288116.45	1292198.16
fnl4461_0100	548909.01	550841.16	552827.55	554682.30	551331.50	553059.70	555654.67
fnl4461_0250	335888.87	336353.60	336579.34	336816.96	336583.34	336889.08	337602.39
fnl4461_0500	224662.49	224719.86	224847.41	224977.65	224913.95	225137.31	225640.28
fnl4461_1000	145862.38	145878.78	145900.40	145935.34	145931.93	146142.23	146746.22
p3038_600	122711.17	122718.26	122750.20	122785.79	122747.37	122807.42	122894.58
p3038_700	109677.30	109681.16	109702.90	109738.74	109692.67	109727.04	109779.24
p3038_800	100064.94	100086.06	100102.13	100147.74	100065.67	100119.52	100198.22
p3038_900	92310.09	92317.52	92325.26	92351.77	92309.87	92332.48	92408.69
p3038_1000	85854.05	85854.67	85857.88	85861.34	85856.13	85864.52	85878.44
sjc1	17288.99	17288.99	17288.99	17288.99	17288.99	17288.99	17288.99
sjc2	33270.94	33270.94	33301.47	33330.42	33270.94	33273.19	33293.40
sjc3a	45335.16	45335.16	45335.16	45335.16	45335.16	45335.16	45335.16
sjc3b	40635.90	40635.90	40635.90	40635.90	40635.90	40635.90	40635.90
sjc4a	61925.51	61925.51	61925.51	61925.51	61925.51	61925.51	61925.51
sjc4b	52458.02	52458.02	52459.08	52461.55	52458.02	52458.37	52461.55

**Table 6:** Details results for GIHH and LAST-RL. The first column lists the instances, and the second column reports the best-known solutions. Columns 3–5 show the minimum, average, and maximum values for GIHH, while the last three columns report the same statistics for LAST-RL

instance	bks	GIHH			LAST−RL		
		min	avg	max	min	avg	max
lin318_005	180281.20	180281.21	180282.26	180282.38	180281.21	180281.33	180282.38
lin318_015	88901.56	88901.56	88901.56	88901.56	88901.56	88901.56	88901.56
lin318_040	47988.38	47988.38	47988.38	47988.38	47988.38	47994.85	48053.12
lin318_070	32198.64	32198.64	32198.64	32198.64	32198.64	32199.58	32208.07
lin318_100	22942.69	22942.69	22942.69	22942.69	22942.69	22942.69	22942.69
ali535_005	9956.77	9956.77	9956.77	9956.77	9956.77	9956.77	9956.77
ali535_025	3695.15	3695.15	3703.55	3717.15	3695.15	3697.65	3703.83
ali535_050	2461.41	2460.93	2466.18	2482.94	2462.65	2469.51	2480.18
ali535_100	1438.42	1440.33	1442.91	1445.89	1437.93	1445.05	1453.90
ali535_150	1032.28	1029.65	1032.78	1037.44	1030.89	1035.12	1041.89
u724_010	181782.96	181776.99	182139.78	182562.16	181776.99	181792.97	181830.83
u724_030	95034.01	95034.01	95084.96	95218.91	95034.01	95074.65	95110.85
u724_075	54735.05	54735.05	54739.19	54776.47	54735.05	54739.19	54776.47
u724_125	38976.76	38976.77	38979.05	38999.56	38976.77	38987.25	39018.40
u724_200	28079.97	28079.97	28079.97	28079.97	28079.97	28079.97	28079.97
rl1304_010	2146484.10	2150973.72	2155222.04	2165824.35	2147899.63	2152288.67	2159525.43
rl1304_050	802283.41	802280.47	803593.18	805633.59	802360.29	804614.82	807217.75
rl1304_100	498090.74	498090.73	498222.30	498506.63	498188.71	498486.02	498824.73
rl1304_200	276977.60	276977.61	276996.98	277075.79	277033.42	277134.02	277265.71
rl1304_300	191224.85	191224.85	191232.40	191275.90	191224.84	191269.66	191368.57
pr2392_020	2235376.73	2230374.00	2238816.43	2247604.15	2229510.95	2231574.48	2235385.97
pr2392_075	1092294.02	1090255.14	1093170.40	1096288.21	1091609.57	1094454.10	1095877.08
pr2392_150	711111.25	711093.89	711558.00	712874.67	711093.89	711587.95	712042.26
pr2392_300	458145.29	458216.44	458454.14	458778.70	458214.77	458358.47	458689.38
pr2392_500	316042.97	316078.39	316352.24	316646.15	316107.78	316364.82	317128.22
fnl4461_0020	1283536.73	1286406.93	1288679.84	1292026.52	1284196.97	1285429.99	1286938.27
fnl4461_0100	548909.01	551589.22	554055.43	556260.92	552022.72	552977.29	553972.35
fnl4461_0250	335888.87	336520.68	337088.74	337863.56	336566.19	337641.73	338707.17
fnl4461_0500	224662.49	224815.50	225003.32	225281.43	224978.71	225172.96	225296.88
fnl4461_1000	145862.38	145909.40	145937.18	145959.97	145947.33	146033.58	146139.88
p3038_600	122711.17	122742.61	122788.40	122831.20	122747.40	122818.35	122920.39
p3038_700	109677.30	109693.76	109726.94	109813.20	109685.14	109799.99	110096.67
p3038_800	100064.94	100085.21	100108.57	100163.64	100093.84	100139.83	100198.82
p3038_900	92310.09	92311.91	92322.41	92337.33	92320.51	92346.14	92379.19
p3038_1000	85854.05	85855.02	85862.25	85875.30	85856.30	85873.71	85920.33
sjc1	17288.99	17288.99	17288.99	17288.99	17288.99	17288.99	17288.99
sjc2	33270.94	33270.94	33270.94	33270.94	33270.94	33275.43	33293.40
sjc3a	45335.16	45335.16	45335.16	45335.16	45335.16	45335.16	45335.16
sjc3b	40635.90	40635.90	40635.90	40635.90	40635.90	40635.90	40635.90
sjc4a	61925.51	61925.51	61925.51	61925.51	61925.51	61925.51	61925.51
sjc4b	52458.02	52458.02	52458.02	52458.02	52458.02	52458.73	52461.55

**Table 7:** New best solutions reported by the proposed techniques

instance	new best solution
ali535_050	$2\,460.81$
ali535_100	$1\,434.08$
ali535_150	$1\,029.46$
u724_010	$181\,776.99$
rl1304_050	$802\,280.47$
pr2392_020	$2\,229\,510.95$
pr2392_075	$1\,090\,120.88$
pr2392_150	$711\,039.65$
XMC10150_100	$178\,932.85$

## Footnotes

1 http://www-usr.inf.ufsm.br/~stefanello/instances/CPMP/

2 https://github.com/phil85/GB21-MH
